# A Highly-sensitized Response of B-type Natriuretic Peptide to Cardiac Ischaemia Quantified by Intracoronary Pressure Measurements

**DOI:** 10.1038/s41598-020-59309-4

**Published:** 2020-02-12

**Authors:** Ryosuke Itakura, Yasunori Inoue, Kazuo Ogawa, Tomohisa Nagoshi, Kosuke Minai, Takayuki Ogawa, Makoto Kawai, Michihiro Yoshimura

**Affiliations:** Division of Cardiology, Department of Internal Medicine, The Jikei University School of Medicine, 3-25-8 Nishi-shinbashi, Minato-ku, Tokyo, 105-8461 Japan

**Keywords:** Ischaemia, Ischaemia

## Abstract

B-type natriuretic peptide (BNP) secretion is stimulated by cardiac dysfunction. However, it is unclear how finely myocardial ischaemia contributes to BNP secretion and whether increases in BNP secretion contribute to coronary vasodilation. This study investigated the direct interaction between plasma BNP levels and cardiac ischaemia using the baseline distal-to-aortic pressure ratio (Pd/Pa). We examined the baseline Pd/Pa and fractional flow reserve (FFR) in 167 patients with intermediate coronary stenosis. The plasma BNP level appeared to be associated with the baseline Pd/Pa in the study population, and this association appeared to become clear only in patients with an FFR ≤ 0.80. To examine the effect of the baseline Pd/Pa on the BNP level in these patients, structural equation modeling (SEM) was performed. The baseline Pd/Pa significantly affected the BNP level (β: −0.37, p = 0.003) and the left ventricular ejection fraction (β: 0.43, p = 0.001). To examine the role of BNP in coronary vasodilation, we proposed another path model using a novel value obtained by dividing the FFR by the baseline Pd/Pa (FFR/baseline Pd/Pa) as an index of the hyperaemic response. The BNP level significantly affected the FFR/baseline Pd/Pa (β: 0.48, p = 0.037). This study demonstrated that BNP finely responded to an exacerbation of cardiac ischaemia and that increases in BNP secretion effectively ameliorated coronary vasoconstriction.

## Introduction

B-type natriuretic peptide (BNP) is secreted mainly by the ventricles in heart failure, whereas normal atria secrete A-type natriuretic peptide (ANP) as well as BNP^1–5^. ANP and BNP have a wide range of biological effects; for instance, they induce vasodilation and natriuresis and inhibit the renin-angiotensin aldosterone system (RAAS) and the sympathetic nervous system^6,7^. Plasma BNP is elevated in heart failure caused by various heart diseases, including ischaemic heart disease (IHD)^8–10^.

Previous reports have shown that myocardial hypoxia associated with a reduction in coronary blood flow increases cardiac BNP expression^11^. Moreover, the BNP level is elevated during early ischaemia, and an elevated BNP level is a significant risk factor for poor short-term and long-term prognoses^12^. Increases in the plasma BNP level are considered a compensatory response of the heart to ischaemia, because several reports have shown that BNP has a vasodilatory effect on the coronary artery system in humans^13,14^. However, it is still unclear how finely myocardial ischaemia itself contributes to BNP secretion and whether increases in BNP secretion actually induce vasodilation as a counter-adaptation. A precise analysis of the relationship between cardiac ischaemia and BNP secretion is the remaining action assignment.

Coronary artery pressure wires are widely used in the clinic to assess the degree of coronary stenosis-induced myocardial ischaemia^15,16^. Pressure wires can be used to measure the fractional flow reserve (FFR), which requires the induction of maximal hyperaemia by drug administration, and the baseline distal-to-aortic pressure ratio (Pd/Pa), which does not require maximal hyperaemia^17^. The Fractional Flow Reserve Versus Angiography for Multivessel Evaluation (FAME) study confirmed that FFR-guided percutaneous coronary intervention (PCI) was a safe long-term treatment for epicardial coronary stenosis;^18^ therefore, using the FFR to determine the need for PCI is acceptable. However, the baseline Pd/Pa is considered a more comprehensive index of coronary circulatory physiology than the FFR^19^. The coronary arterial tone is probably augmented in most patients with atherosclerosis. Therefore, we surmised that the baseline Pd/Pa would be a sensitive and useful index for evaluating coronary ischaemia.

Herein, we devised a new investigative method to examine the relationship between the BNP level and coronary ischaemia and performed simultaneous measurements of the plasma BNP level and the baseline Pd/Pa in patients with intermediate coronary artery stenosis.

However, studies of this type are faced with another degree of intractableness. Many confounding factors can affect the plasma BNP level and baseline Pd/Pa. When confounders are present, the results do not reflect the actual relationship between the studied variables. Confounding variables are variables that are either positively or negatively correlated with both the dependent and independent variables, and bias caused by confounding variables can be difficult to prevent if multiple potential confounding variables are present or the study population lacks a sufficient size. SEM plays an important role in enabling researchers to understand how relationships between observed variables may develop. This analysis is useful for exploratory and explanatory factor analyses and can also be performed to assess relationships between variables in cases in which confounding bias may be present. In this study, we tried to propose a path model based on SEM to explain a complex phenomenon.

## Results

### Characteristics of the study population

The baseline characteristics, angiographic measurements and catheterization data for the study population are shown in Table 1. A total of 167 patients were investigated in this study. The median age of the study population was 68.0 [63.0–75.0] years, the median percent diameter stenosis (%DS) was 48.1 [40.0–53.2] %, the median FFR was 0.83 [0.76–0.88], and the median baseline Pd/Pa was 0.94 [0.92–0.97]. Overall, the study population displayed coronary stenosis of intermediate severity.

**Table 1:** Clinical characteristics.

Characteristics (n = 167)	Number (%) or Median [interquartile range]
FFR ≤ 0.80	64 (38.3)
FFR > 0.80	103 (61.7)
Baseline Pd/Pa	0.94 [0.92–0.97]
FFR	0.83 [0.76–0.88]
Age (years old)	68.0 [63.0–75.0]
Gender; Male	143 (85.6)
Body mass index (kg/m²)	24.5 [22.3–26.7]
*Blood data*	
BNP (pg/ml)	38.7 [17.1–131.5]
Hb (g/dl)	13.3 [12.1–14.5]
HbA1c (%)	6.2 [5.7–6.8]
s-Cr (mg/dl)	0.87 [0.74–1.06]
UA (mg/dl)	5.9 [4.8–6.6]
Fasting IRI (µU/ml)	6.6 [4.7–10.4]
HOMA-IR	1.8 [1.1–2.9]
*Heart disease*	
Cardiomyopathy	11 (6.6)
Valvular disease	5 (3.0)
AF	9 (5.4)
*Coronary artery disease risk factors*	
Hypertension	144 (86.2)
Diabetes mellitus	84 (50.3)
Dyslipidaemia	133 (79.6)
Current + past smokers	114 (68.3)
HD & CAPD	17 (10.2)
*Lesion location*	
LAD	114 (68.3)
LCX	20 (12.0)
RCA	31 (18.6)
LMT	2 (1.2)
*Vessel disease*	
0-VD	77 (46.1)
1-VD	61 (36.5)
2-VD	24 (14.4)
3-VD	5 (3.0)
*QCA*	
% Diameter stenosis, (%)	48.1 [40.0–53.2]
*Haemodynamic variables*	
LVEF (%)	61.5 [55.9–66.0]
LVEDP (mmHg) (at preLVG)	13.0 [10.0–17.0]
SBP (mmHg)	133.0 [116.0–156.0]
DBP (mmHg)	69.0 [61.0–78.0]
Heart rate (beat/min)	68.0 [59.0–77.0]
*Medications*	
Calcium-channel blockers	104 (62.3)
ACE inhibitors/Angiotensin receptor blockers	112 (67.1)
Nitrates/Nicorandil	59 (35.3)
Nicorandil	22 (13.2)
Beta-blockers	83 (49.7)
Aldosterone blocker	10 (6.0)
Statins	111 (66.5)
Diuretics	136 (16.2)
Oral hypoglycaemia agents	43 (25.7)

FFR, fractional flow reserve; Pd/Pa, distal-to-aortic pressure ratio; BNP, B-type natriuretic peptide; Hb, haemoglobin; HbA1c, haemoglobin A1c; s-Cr, serum creatinine; UA, serum uric acid; IRI, immunoreactive insulin; HOMA-IR, homeostasis model assessment of insulin resistance; AF, atrial fibrillation; HD, haemodialysis; CAPD, continuous ambulatory peritoneal; LAD, left anterior descending; LCX, left circumflex coronary artery; RCA, right coronary artery; LMT, left main trunk; VD, vessel disease; LVEF, left ventricular ejection fraction; LVEDP, left ventricular end-diastolic pressure; CI, cardiac index; SBP, systolic blood pressure; DBP, diastolic blood pressure.

### Associations between the baseline Pd/Pa and FFR

Single regression analysis showed that the baseline Pd/Pa was clearly positively correlated with the FFR (r = 0.74, p < 0.001) in the study population. Nevertheless, the scatter plot appeared to spread as the baseline Pd/Pa and FFR decreased (Supplementary Fig. S1).

### Associations between the baseline Pd/Pa and LogBNP levels

**Figure 1:**
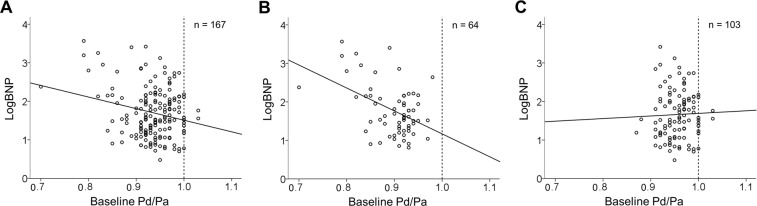
Associations between the baseline Pd/Pa and LogBNP levels. The baseline Pd/Pa and LogBNP levels are represented as scatter plots. The lines shown here simply represent the relationships with no added statistical analysis. **(A)** Entire study population (n = 167). (**B**) FFR ≤ 0.8 group (n = 64). (**C**) FFR > 0.8 group (n = 103). FFR, fractional flow reserve; LogBNP, logarithmic B-type natriuretic peptide; Pd/Pa, distal-to-aortic pressure ratio.

In the plotted graph (Fig. 1), the baseline Pd/Pa appeared to be associated with the LogBNP in all cases (n = 167). A tendency for negative relationships was observed; the relationship appeared to be strong in the FFR ≤ 0.8 group (n = 64) but weak in the FFR > 0.8 group (n = 103). In other words, in the group with an FFR ≤ 0.8, we judged that the relationship between the baseline Pd/Pa and LogBNP was remarkable. Therefore, we proceeded to the next full-scale study (covariance structural analysis) using this group.

### Concept of the proposed path model A: Investigation of the impact of the baseline Pd/Pa on the plasma BNP level

**Figure 2:**
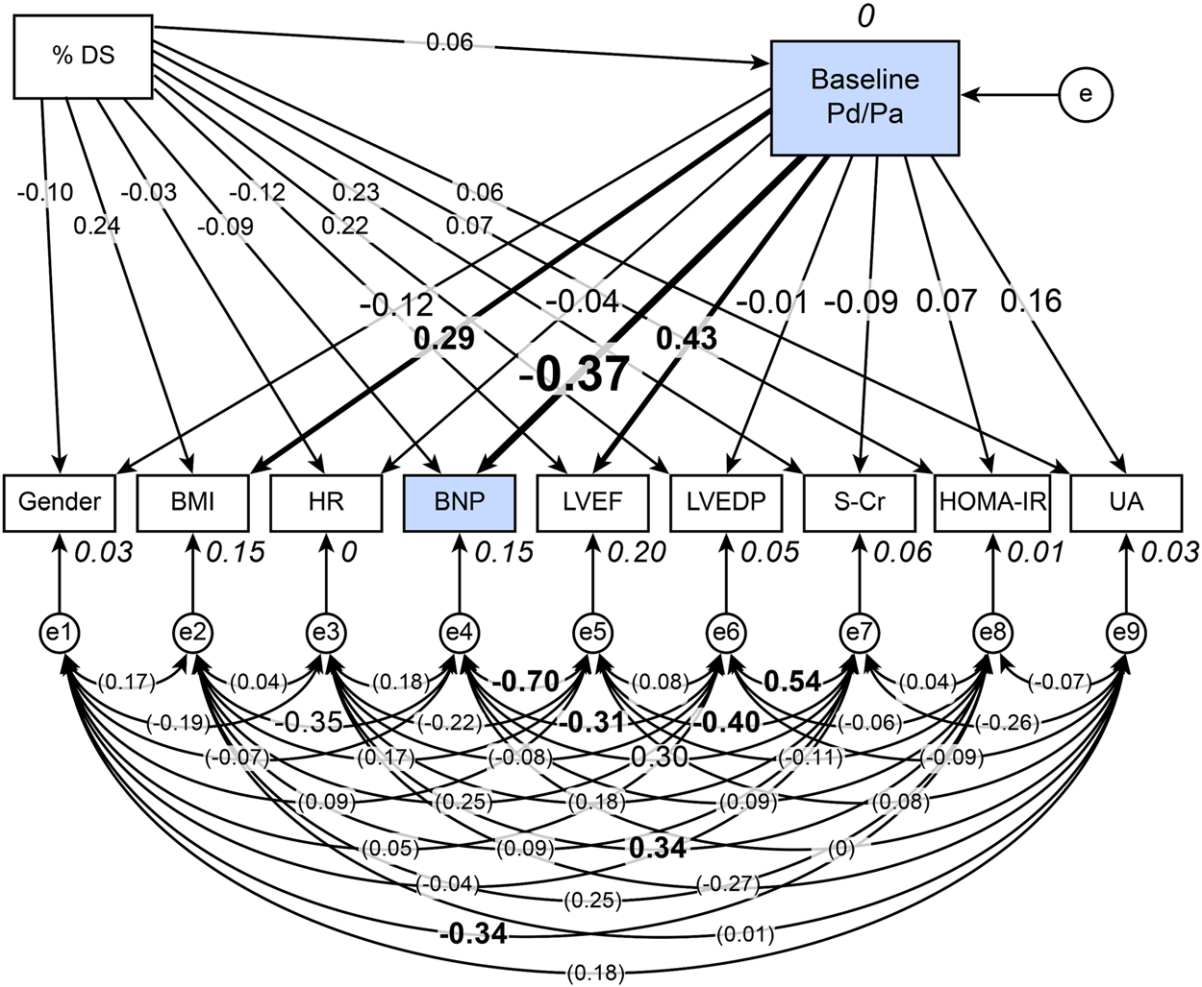
Path model A: Investigation of the impact of the baseline Pd/Pa on the plasma BNP levels in the FFR ≤ 0.8 group (n = 64). This path has a coefficient showing the standardized coefficient for a regressing independent variable on the dependent variable of the relevant path. These variables indicate standardized regression coefficients and squared multiple correlations [italicized capitalized variables]. HR, heart rate; LVEDP, left ventricular end-diastolic pressure; LVEF, left ventricular ejection fraction; %DS, % diameter stenosis.

To clarify the possible impact of the baseline Pd/Pa on the plasma BNP level, we conducted a path model analysis based on covariant structure analysis. The proposed theoretical path model is shown in Fig. 2. The path model featured a hierarchical structure including gender, body mass index (BMI), heart rate (HR), left ventricular ejection fraction (LVEF), left ventricular end-diastolic pressure (LVEDP), serum creatinine (S-Cr), homeostasis model assessment of insulin resistance (HOMA-IR), serum uric acid (UA), %DS and BNP. Gender, BMI, HR, LVEF, and LVEDP are widely known to be major determinants of BNP^20–22^. Since we recently reported that BNP might be related to HOMA-IR and UA, these two factors were also included in the path diagram^23–25^. We excluded patients with arterial fibrillation (AF) (n = 1) and those receiving insulin therapy (n = 8) to reduce possible bias. The correlations between any two of these factors, which might have been confounders, were indicated by two-way arrows. The paths between variables were drawn from the independent variables to the dependent variables with directional arrows for every regression model (i.e., from the baseline Pd/Pa to gender, BMI, HR, BNP, LVEF, LVEDP, S-Cr, HOMA-IR and UA). In this path model, the %DS was positioned on top of the other factors because the degree of organic stenosis could essentially affect every other parameter included in the model.

### The path model A results

The precise statistical analysis results are shown in Fig. 2 and Table 2. The baseline Pd/Pa significantly affected the BNP level (standardized regression coefficient: β: −0.37, p = 0.003), BMI (β: 0.29, p = 0.019) and LVEF (β: 0.43, p = 0.001).

**Table 2:** The results of the path model described in Fig. 2.

Clinical factor		Estimate	Standard error	Test statistic	P value	Standard regression coefficient		
						Direct effect	Indirect effect	Total effect
Baseline Pd/Pa (R^2^ = 0)	–>Gender	−0.59	0.63	−0.93	0.352	−0.12	0	−0.12
	–>BMI	21.72	9.24	2.35	0.019	0.29	0	0.29
	–>HR	−10.97	35.20	−0.31	0.755	−0.04	0	−0.04
	–>BNP	−4812.42	1643.79	−2.93	0.003	−0.37	0	−0.37
	–>LVEF	108.65	33.32	3.26	0.001	0.43	0	0.43
	–>LVEDP	−1.47	24.81	−0.06	0.953	−0.01	0	−0.01
	–>S-Cr	−4.32	6.32	−0.68	0.494	−0.09	0	−0.09
	–>HOMA-IR	2.19	4.56	0.48	0.631	0.07	0	0.07
	–>UA	4.40	3.62	0.44	0.225	0.16	0	0.16
%DS	–>Gender	−0.00	0	−0.77	0.439	−0.10	−0.01	−0.11
	–>BMI	0.09	0.05	1.93	0.053	0.24	0.02	0.26
	–>HR	−0.04	0.17	−0.22	0.824	−0.03	−0.03	−0.03
	–>BNP	−5.80	8.01	−0.72	0.469	−0.09	−0.02	−0.11
	–>LVEF	−0.15	0.16	−0.90	0.368	−0.12	0.02	−0.10
	–>LVEDP	0.18	0.12	1.51	0.132	0.22	0	0.21
	–>S-Cr	0.06	0.03	1.79	0.073	0.23	−0.01	0.23
	–>HOMA-IR	0.01	0.02	0.53	0.595	0.07	0.00	0.07
	–>UA	0.01	0.02	0.44	0.662	0.06	0.01	0.07
	–>Baseline Pd/Pa	0	0	0.42	0.674	0.06	0	0.06

The results (direct, indirect, and total effects) of the path model theoretical analysis to identify clinical factors influencing each other. R2: squared multiple correlations.

Pd/Pa, distal-to-aortic pressure ratio; BNP, B-type natriuretic peptide; %DS, % diameter stenosis; BMI, body mass index; HR, heart rate; LVEF, left ventricular ejection fraction; LVEDP, left ventricular end-diastolic pressure; S-Cr, serum creatinine; HOMA-IR, homeostasis model assessment of insulin resistance; UA, serum uric acid.

### Concept of the proposed path model B: Investigation of the impact of the BNP level on the FFR/baseline Pd/Pa ratio

**Figure 3:**
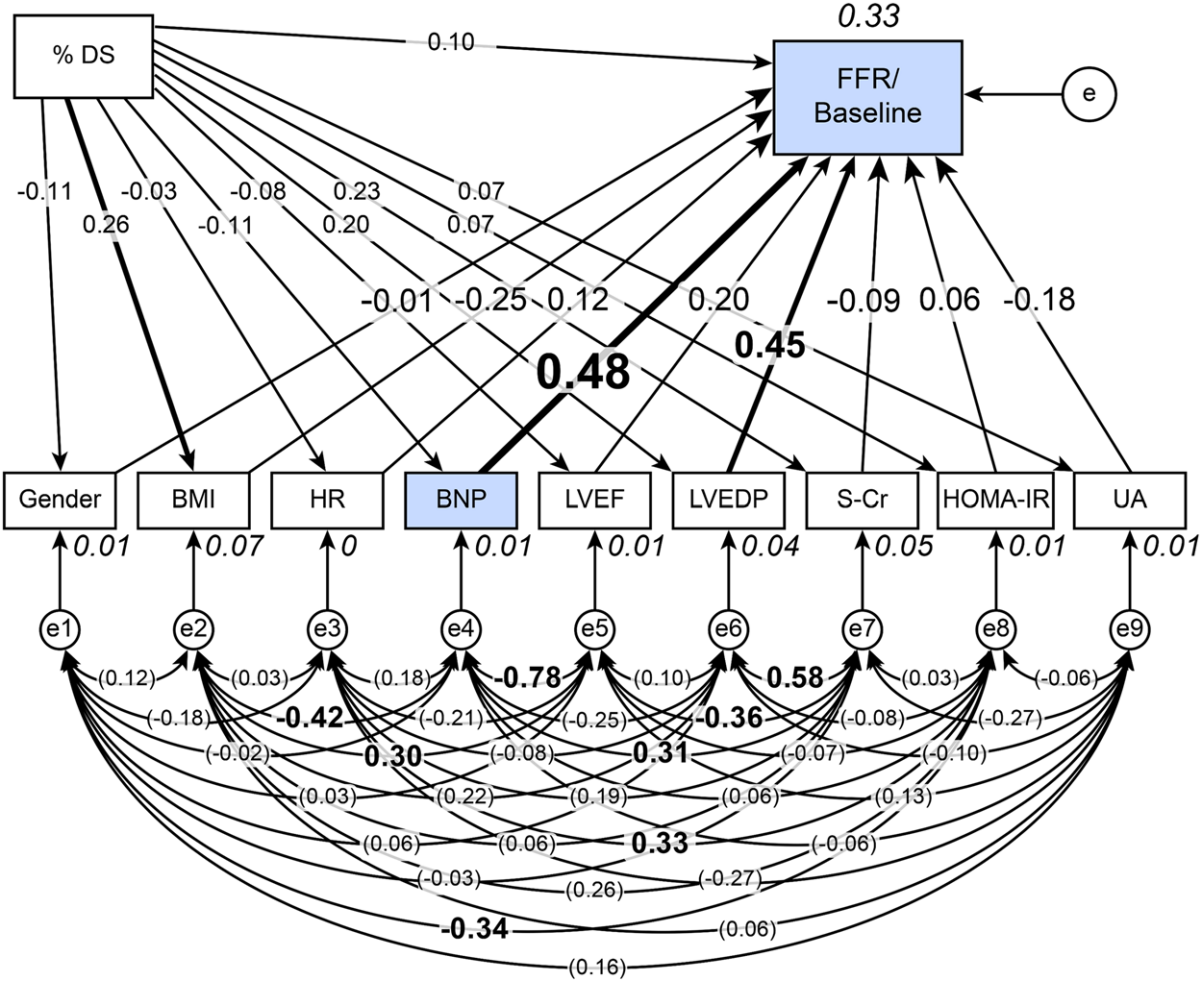
Path model B: Investigation of the impact of BNP on the FFR/baseline Pd/Pa ratio in the FFR ≤ 0.8 group (n = 64). This path has a coefficient showing the standardized coefficient for a regressing independent variable on the dependent variable of the relevant path. These variables indicate standardized regression coefficients and squared multiple correlations [italicized capitalized variables].

To clarify the impact of the BNP level on the FFR/baseline Pd/Pa ratio, we conducted another path analysis. The proposed theoretical path model is shown in Fig. 3. The path model featured a hierarchical structure including gender, BMI, HR, LVEF, LVEDP, S-Cr, HOMA-IR, UA, %DS and BNP. We excluded patients with AF (n = 1) and those receiving insulin therapy (n = 8). The correlations between any two of these factors were indicated using two-way arrows. The paths between variables were drawn from the independent variables to the dependent variables with directional arrows for every regression model (i.e., from gender, BMI, HR, BNP, LVEF, LVEDP, S-Cr, HOMA-IR, UA and %DS to the FFR/baseline Pd/Pa ratio). As shown for path model A, in this model, the %DS was positioned above the other factors.

### The path model B results

The statistical analysis results are shown in Fig. 3 and Table 3. The FFR/baseline Pd/Pa ratio was significantly affected by the BNP level (β: 0.48, p = 0.037) and LVEDP (β: 0.45, p = 0.022).

**Table 3:** The results of the path model described in Fig. 3.

Clinical factor		Estimate	Standard error	Test statistic	P value	Standard regression coefficient		
						Direct effect	Indirect effect	Total effect
FFR/Baseline Pd/Pa (R^2^ = 0.33)	<–Gender	0	0.03	−0.08	0.939	−0.1	0	−0.1
	<–BMI	0	0	−1.70	0.088	−0.25	0	−0.25
	<–HR	0	0	0.91	0.361	0.12	0	0.12
	<–BNP	0	0	2.08	0.037	0.48	0	0.48
	<–LVEF	0	0	0.91	0.364	0.20	0	0.20
	< –LVEDP	0	0	2.29	0.022	0.45	0	0.45
	<–S-Cr	0	0.01	−0.45	0.656	−0.09	0	−0.09
	<–HOMA-IR	0	0.01	0.43	0.667	0.06	0	0.06
	<–UA	−0.01	0.01	−1.44	0.149	−0.18	0	−0.18
%DS	–> Gender	0	0	−0.82	0.412	−0.11	0	−0.11
	–> BMI	0.09	0.05	1.97	0.048	0.26	0	0.26
	–> HR	−0.04	0.17	−0.24	0.810	−0.03	0	−0.03
	–> BNP	−7.12	8.60	−0.83	0.407	−0.11	0	−0.11
	–> LVEF	−0.10	0.19	−0.52	0.604	−0.08	0	−0.08
	–> LVEDP	0.17	0.12	1.40	0.162	0.20	0	0.20
	–> S-Cr	0.05	0.03	1.75	0.081	0.23	0	0.23
	–> HOMA-IR	0.01	0.02	0.55	0.585	0.07	0	0.07
	–> UA	0.01	0.02	0.50	0.617	0.07	0	0.07
	–> FFR/Baseline Pd/Pa	0	0	0.79	0.428	0.10	−0.07	0.03

The results (direct, indirect, and total effects) of the path model theoretical analysis to identify clinical factors influencing each other. R2: squared multiple correlations.

Pd/Pa, distal-to-aortic pressure ratio; BNP, B-type natriuretic peptide; FFR, fractional flow reserve; %DS, % diameter stenosis; BMI, body mass index; HR, heart rate; LVEF, left ventricular ejection fraction; LVEDP, left ventricular end-diastolic pressure; S-Cr, serum creatinine; HOMA-IR, homeostasis model assessment of insulin resistance; UA, serum uric acid.

### Association between the FFR/baseline Pd/Pa ratio and the LogBNP level

**Figure 4:**
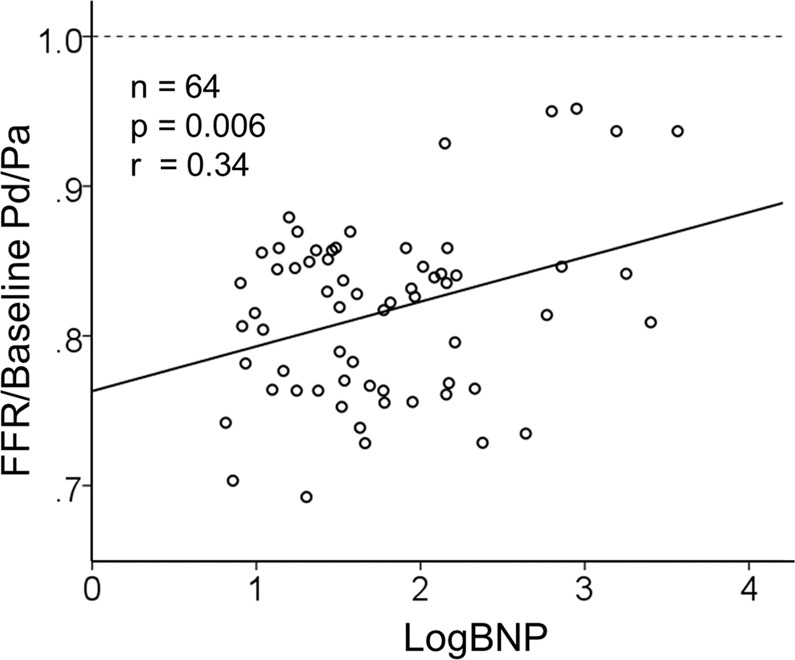
Association between the FFR/baseline Pd/Pa ratio and LogBNP levels in the FFR ≤ 0.8 group (n = 64). The FFR/baseline Pd/Pa ratio and LogBNP levels are represented as scatter plots. The solid black line indicates the regression curve for the fitted logarithmic equation.

Single regression analysis showed that the LogBNP was significantly positively correlated with the FFR/baseline Pd/Pa ratio (r = 0.34, p = 0.006) in the FFR ≤ 0.8 group (Fig. 4). As the LogBNP level increased, the FFR/baseline Pd/Pa ratio approached but fell short of 1.0. We investigated frailty by removing a few outliers in Fig. 4 (n = 63 with omission of 1 case). However, no essential change was found from the results obtained for all patients (data not shown).

### Investigation of the validity of an FFR cut-off of 0.8 using BNP values

An FFR value of 0.8 is a widely accepted cut-off and was used for that purpose in this study^18^. Nonetheless, we thought that it would be possible to examine whether this cut-off value is appropriate from the viewpoint of BNP using the current data. Therefore, we investigated the validity of an FFR cut-off value of 0.8 using the association between baseline Pd/Pa and LogBNP levels. The slope of the regression line in Fig. 1C was further examined in response to varying FFR cut-off values; we searched for the cut-off value at which the slope of regression line between baseline Pd/Pa and LogBNP levels became 0. In particular, the slope of the regression line was examined at a range of cut-off values that increased from 0.66 to 0.8 in steps of 0.1: cut-off value = 0.66 (n = 161), 0.67 (n = 160), 0.68 (n = 157), 0.69 (n = 153), 0.70 (n = 151), 0.71 (n = 144), 0.72 (n = 141), 0.73 (n = 135), 0.74 (n = 131), 0.75 (n = 128), 0.76 (n = 124), 0.77 (n = 118), 0.78 (n = 114), 0.79 (n = 107), and 0.80 (n = 103). These values were plotted as shown in Supplementary Figure S2, and the regression lines of these plots were drawn. The FFR at which the slope of the regression line became 0 was found to be 0.795. This value is very close to 0.8, suggesting that an FFR of 0.8 is appropriate from the viewpoint of BNP. Of course, this result was obtained by simply drawing regression lines, and the true meaning of this result could be examined by other methods in the future.

## Discussion

**Figure 5:**
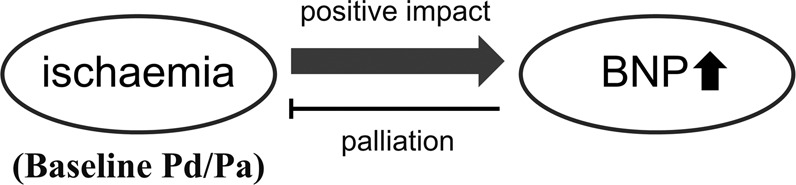
Schematic illustration. A schematic illustration of our findings.

The major findings of this study were as follows: 1) cardiac ischaemia, as represented by the nonhyperaemic baseline Pd/Pa, had a causative impact on the plasma BNP level, suggesting the presence of a fine regulatory system of BNP by cardiac ischaemia; 2) BNP had a positive impact on the FFR/baseline Pd/Pa ratio, suggesting that a vasodilating action of BNP occurred in a compensatory manner; and 3) increasing BNP secretion was effective for palliation of the coronary arterial tonus, but the produced quantity of BNP was still inadequate for full coronary vasodilatation even in patients with high plasma BNP levels. A schematic illustration of our findings is shown in Fig. 5.

In the first phase of this study, we noted that the plasma BNP level appeared to be associated with the baseline Pd/Pa in the study population. This association appeared to become clear only in patients with coronary stenosis with an FFR of 0.80 or less, which is widely recognized as being suggestive of an ischaemic circumstance^18^. This result indicated a possible direct relationship between cardiac ischaemia and BNP secretion. Then, we tried to determine a possible effect of cardiac ischaemia on the plasma BNP level using SEM. Since myocardial stretch or haemodynamic deterioration stimulates BNP secretion, many confounding factors exist with the plasma BNP level, such as LVEDP and LVEF. To account for possible confounding bias, we proposed a path model and revealed a possible effect of cardiac ischaemia on the plasma BNP level. Importantly, the contribution was fairly strong, because the standard regression coefficient was extremely high (β: −0.37), suggesting the presence of an exquisite BNP regulatory system driven by cardiac ischaemia.

Previous reports suggested that BNP could affect coronary vasodilation at ischaemic sites in a compensative manner^13,14^. Thus, we investigated the possible direct effect of BNP on palliation of the arterial tonus. To quantify this effect, we introduced a novel measure (the FFR/baseline Pd/Pa ratio), which served as an index of the hyperaemic response. Then, another path model was proposed for this analysis, and we successfully found a potential effect of BNP on coronary vasodilation as a compensatory phenomenon.

To reveal the power of secreted BNP for vasodilation, we examined the relationship between the BNP level and the FFR/baseline Pd/Pa ratio using regression analysis. The analysis showed a significant positive correlation between the BNP level and the FFR/baseline Pd/Pa ratio; importantly, as the BNP level increased, the FFR/baseline Pd/Pa ratio approached but still fell short of 1.0. This result suggested that the level of BNP secretion induced by ischaemia was insufficient to induce full coronary vasodilation even in patients with relatively high plasma BNP levels.

Speculation about the action sites of BNP in the coronary arteries is important because BNP can act at the levels of the epicardial coronary arteries and the coronary microvessels. If BNP acts on resistant coronary arteries, it may have promising flow-dependent coronary vasodilation-inducing properties. In our previous study, we reported that BNP had vasodilatory effects on the coronary artery system in humans and that the sensitivity of coronary resistance vessels to BNP was low compared with that of resistance vessels of the systemic circulation^13^. Currently, we believe that BNP acts mainly on both the epicardial coronary arteries and the coronary microvessels.

Natriuretic peptides exert direct vasodilatory effects by increasing the level of cyclic GMP, which acts as a second messenger^26^. Furthermore, recent studies have noted a possible interaction of natriuretic peptides with NO activity. Interestingly, the natriuretic peptide system, including A- and C-type natriuretic peptides (ANP and CNP), has been reported to be involved in NO activity^27^. ANP restores the attenuated cardioprotective effects in diabetic hearts, possibly due to the increase in endothelial nitric oxide synthesis (eNOS) expression and subsequent increase in NO activity^27^. Moreover, CNP induces vascular relaxation by endothelium-dependent and endothelium-independent mechanisms^28,29^. Although more precise human studies are required, the interaction of natriuretic peptides and NO activity should be closely linked; natriuretic peptides would support the impaired NO activity in diseased vessels and the attenuated vascular tonus.

The results of this study suggest that the plasma BNP level increases in response to cardiac ischaemia and that increases in the plasma BNP level attenuate the coronary arterial tonus; however, the level at which BNP is secreted is insufficient to induce full coronary vasodilation. These results indicate that natriuretic peptides may be useful as a therapy for IHD in the future. Infusions of synthetic ANP (carperitide) and BNP (nesiritide) would be effective in inducing coronary vasodilation. Indeed, we previously reported that ANP and BNP were effective in suppressing coronary artery spasm during hyperventilation tests^30,31^. Additionally, chronic ANP treatment has been reported to ameliorate hypertension and end-organ damage in the kidney by reducing oxidative stress, increasing systemic NO activity levels, and diminishing the collagen content and apoptosis in both sexes^32^. Moreover, angiotensin receptor-neprilysin inhibitor (ARNi) is expected to contribute to these outcomes^33^. However, a matter of paramount importance is controlling obesity through diet and exercise because the plasma natriuretic peptide levels are comparatively low in patients with obesity, and their influence increases as the degree of obesity becomes more severe^23,34^. Importantly, we also reported that the plasma BNP levels were relatively low in patients with chronic IHD compared with those of non-IHD patients^35^ and that the low reactivity of BNP could play a causative role in IHD^36^. We believe that treatment of obesity will be beneficial for preventing IHD in part by increasing endogenous natriuretic peptide levels.

Most of the stenotic coronary arterial lesions encountered herein were single moderate stenotic lesions, but multi-vessel coronary artery disease was infrequently encountered. BNP secretion may be affected by the presence of other stenotic vessels. However, we performed the same analyses for patients with single moderate stenotic lesions (n = 42); the results were similar, and the baseline Pd/Pa had the same impact on the BNP level in the single-vessel group (p = 0.002) (data not shown). Additionally, the %DS was included in path models A and B; however, we surmised that a simpler path model might be sufficient to satisfy the objectives of the current study. Thus, we performed additional analyses using path models in which the %DS was not included. The results of these analyses were similar to those of the analyses performed using the path models in which the %DS was included (data not shown). Moreover, because the baseline Pd/Pa might be influenced by micro-circulatory function^19^, variables such as diabetes mellitus, hypertension, dyslipidaemia and the smoking status could affect the measurement. We also performed a multiple regression analysis of factors to determine the baseline Pd/Pa using the variables BNP, diabetes mellitus, hypertension, dyslipidaemia, and smoking status in patients with an FFR ≤ 0.80. As a result, the BNP levels inversely correlated with the baseline Pd/Pa (p = 0.026) (data not shown).

In this study, we examined the relationship between endogenous plasma BNP and the actual ischaemic heart. However, confirming the relationship between plasma BNP and the ischaemic heart may require inducing ischaemia and then observing changes in plasma BNP. In addition, synthetic BNP may need to be administered exogenously to test for coronary artery dilatation. These experiments must be planned using animal models.

An FFR value of 0.8 is a widely accepted cut-off. In this study, however, we examined the validity of this cut-off using BNP values. In Supplementary Figure S2, the X-axis represents the FFR cut-off value, and the Y-axis represents the regression coefficient between baseline Pd/Pa and LogBNP levels. We found that the FFR at which the slope of the regression line became 0 was 0.795.

### Points to note on SEM

SEM is a convenient analytical method and can be used to easily estimate parameters, so it is used in a wide range of academic fields. However, the following points should be noted:

1. SEM is an effective analysis method for confirming hypotheses, but the analysts need to examine a sufficient number of hypotheses before and after model construction. By making full use of the analyst’s knowledge, the hypotheses approach the right model. In other words, trial and error is necessary. However, this is also a good aspect of SEM.
2. The next problem concerns cause and effect. For confounding variables (the third variable that affects both the cause and effect variables), the causal relationship of the target must be examined. To further refine the cause and effect, detailed confounding variables need to be considered. Furthermore, to be causal, strictly speaking, priorities must be discussed before the event occurs in terms of the temporal priority at which the causal event occurs. Care must be taken to conclude that there is an exact causal relationship without such a consideration. Therefore, this paper does not necessarily indicate cause and effect. A directed acyclic graph may be effective as an alternative method for strictly discussing causality.

### Study limitation

The present study was associated with some limitations. First, we measured only circulating BNP levels in this study. Ideally, plasma BNP levels in the coronary sinus may be required to clarify more precisely the interaction between the BNP level and cardiac ischaemia. Blood sampling was performed almost immediately before coronary pressure measurement in almost all cases. Therefore, although we do not think that a time lag exists between blood sampling and haemodynamic measurement, the effect of some time lag cannot be completely denied. Second, we used different types of drugs during cardiac catheterization for treatment of IHD. Thus, we could not exclude the possibility that these drugs affected the current results. Third, the ischaemic myocardial area subtended by the stenosis might influence BNP, but we did not consider the myocardial area or lesion locations in our analyses. Ideally, cardiac ischaemia might be measured as oxygen tension, which is the physiological stimulus for cardiomyocytes. However, we did not measure the myocardial oxygen tension in this study. Fourth, the path analysis makes many assumptions. For example, linearity is assumed for a very large number of relationships. Therefore, we may need to build other path models and perform additional analysis.

In this study, in which we simultaneously measured the baseline Pd/Pa and plasma BNP levels, we demonstrated that BNP secretion finely responded to and was increased by cardiac ischaemia. The increase in BNP secretion was effective for palliation of coronary vasoconstriction. However, the level of BNP secretion induced by ischaemia was still inadequate for full coronary dilatation.

## Methods

### Study patients

The study population initially consisted of 1930 patients with IHD who were consecutively admitted to our institutions from March 2014 to July 2016. We performed cardiac catheterization on all patients and completed the required baseline Pd/Pa and FFR measurements in 167 patients^37^. We routinely examined both values to accurately determine each patient’s need for further catheter-based interventions. We excluded patients who required emergent treatment (e.g., patients with acute coronary syndrome) and patients with severe valve diseases because their haemodynamic statuses and biomarker levels were highly variable and did not reflect the values under stable conditions. The study population of 167 patients was divided into two groups according to their FFR values. The FFR ≤ 0.8 group consisted of patients whose FFR was ≤ 0.8, and the FFR > 0.8 group consisted of patients whose FFR was > 0.8. The ethics committee of the Jikei University School of Medicine approved the study protocol (24–355[7121]), and we complied with the routine ethical regulations of our institution. Informed consent was obtained from each patient, and all clinical investigations were conducted in accordance with the principles expressed in the Declaration of Helsinki. We also posted a notice about the study design and contact information at a public location in our institution.

### Measurement of the plasma BNP level

Whole blood (5 ml) was collected in tubes containing potassium EDTA (1 mg/ml of blood), and the plasma BNP levels were determined by enzyme-linked immunosorbent assay (non-extracted) using an antibody against human BNP (Shionogi Co., Ltd., Tokyo, Japan). Blood sampling was performed almost immediately before coronary pressure measurement in almost all cases.

### Catheterization procedure and baseline Pd/Pa and FFR measurements

Coronary angiography was performed via a radial, brachial or femoral artery approach in standard multiple views by experienced operators using 5–7 French guiding catheters without side holes. All patients received a bolus of heparin (at least 2000|IU) before the procedure. A 0.014-inch pressure-monitoring guidewire (PressureWire Aeris, St. Jude Medical, St Paul, MN, USA or Verrata, Volcano Corporation, Rancho Cordova, CA, USA) was externally calibrated and advanced through the guiding catheter to its tip before being placed at a location distal to the stenosis. Subsequently, the Pd and Pa were measured at rest and during drug-induced hyperaemia^38^ as described in further detail below. The nonhyperaemic baseline Pd/Pa and FFR were calculated as a ratio of Pd to Pa at rest and during hyperaemia and reported as the average of ratios measured during 5 cardiac cycles. Adenosine (Trinosin-S inj, Toaeiyo, Tokyo, Japan) or papaverine (Papaverine hydrochloride, Nichiikou, Toyama prefecture, Japan) was used to induce hyperaemia in this study. Adenosine was administered through a peripheral vein at a dose of 150 μg/kg/min, and papaverine was administered via the left coronary artery at a dose of 12 mg and via the right coronary artery at a dose of 8 mg, which induced maximal dilatation within 15 s. Intracoronary isosorbide dinitrate (at least 0.5 mg) was administered before coronary angiography in all patients. In this study, we propose the value obtained by dividing the FFR by the baseline Pd/Pa (FFR/baseline Pd/Pa) as an index of vasodilation. A value of 1 indicated that the coronary arteries were fully dilated even at the basal condition.

### Quantitative coronary angiography

Quantitative coronary angiography was performed by an independent analyser blinded to the FFR data. Using a guiding catheter for calibration and an edge detection system (QAngioXA V.7.1, Medis, Leiden, The Netherlands), the analyser measured the reference vessel diameter and minimum lumen diameter and calculated the %DS.

### Statistical analysis

Continuous variables are expressed as the means ± standard deviations (SDs) or as medians [interquartile range]. To achieve a normal distribution, we log-transformed the BNP levels prior to the analysis. Correlations between two factors were investigated by single regression analysis and expressed as Pearson correlation coefficients. Multiple logistic regression analysis was performed as needed. All statistical analyses were performed using IBM SPSS Statistics version 23.0 (SPSS Inc., Chicago, IL, USA). P values < 0.05 were considered statistically significant. Path analysis based on SEM was used to investigate the relationships among clinical factors in this study population, specifically to survey probable causal effects for the baseline Pd/Pa. The path analysis was performed with IBM SPSS AMOS version 23 (Amos Development Corporation, Meadville, PA, USA). This analysis compares the power among multiple independent variables that confound each other^34^. The obtained structural equation models were tested and confirmed at the P < 0.05 significance level. Briefly, the model defines some hierarchical regression models that determine the Pd/Pa. Paths between variables were drawn from the independent variable to the dependent variables with directional arrows for every regression model (arrowheads on one end only). The presence of a two-way arrow between two variables was indicative of a correlation between these two variables. For every regression, the total variance in the dependent variable was theorized to be affected either by variables that were independent of the model or by extraneous variables. Each path had a standardized coefficient of the regressing independent variable on the dependent variable for the relevant path. The indirect effects were determined by multiplying the path coefficients of these intervening variables.

## Supplementary information

Supplementary information.
