# Supplementary material for: A Highly-sensitized Response of B-type Natriuretic Peptide to Cardiac Ischaemia Quantified by Intracoronary Pressure Measurements
Source: Sci Rep. 2020 Feb 12;10:2403. doi: 10.1038/s41598-020-59309-4 (PMC7015889; doi:10.1038/s41598-020-59309-4)
Supplement: Supplementary file 1 — Supplementary information. [file 41598_2020_59309_MOESM1_ESM.docx]

**Supplementary Information**

**A Highly-sensitized Response of B-type Natriuretic Peptide to Cardiac Ischaemia Quantified by Intracoronary Pressure Measurements**

Ryosuke Itakura, MD, Yasunori Inoue, MD, PhD, Kazuo Ogawa, MD, PhD, Tomohisa Nagoshi, MD, PhD, Kosuke Minai, MD, PhD, Takayuki Ogawa, MD, PhD, Makoto Kawai, MD, PhD, Michihiro Yoshimura, MD, PhD

Division of Cardiology, Department of Internal Medicine, The Jikei University School of Medicine, 3-25-8 Nishi-shinbashi, Minato-ku, Tokyo 105-8461, Japan.

**Address for correspondence**

Corresponding author: Yasunori Inoue, MD, PhD

Division of Cardiology, Department of Internal Medicine, The Jikei University School of Medicine, 3-25-8 Nishi-shinbashi, Minato-ku, Tokyo l05-8461, Japan.

Phone: +81-3-3433-1111

Fax: +81-3-3459-6043

E-mail: [y.inoue@jikei.ac.jp](mailto:y.inoue@jikei.ac.jp)

**Authors’ email addresses**

Ryosuke Itakura, MD [ms04-itakura@jikei.ac.jp](mailto:ms04-itakura@jikei.ac.jp)

Kazuo Ogawa, MD, PhD [oga-n@jikei.ac.jp](mailto:oga-n@jikei.ac.jp)

Tomohisa Nagoshi, MD, PhD [tnagoshi@jikei.ac.jp](mailto:tnagoshi@jikei.ac.jp)

Kosuke Minai, MD, PhD [heart@jikei.ac.jp](mailto:heart@jikei.ac.jp)

Takayuki Ogawa, MD, PhD [takaog39@jikei.ac.jp](mailto:takaog39@jikei.ac.jp)

Makoto Kawai, MD, PhD [cadmk_m@jikei.ac.jp](mailto:cadmk_m@jikei.ac.jp)

Michihiro Yoshimura, MD, PhD [m.yoshimura@jikei.ac.jp](mailto:m.yoshimura@jikei.ac.jp)

**
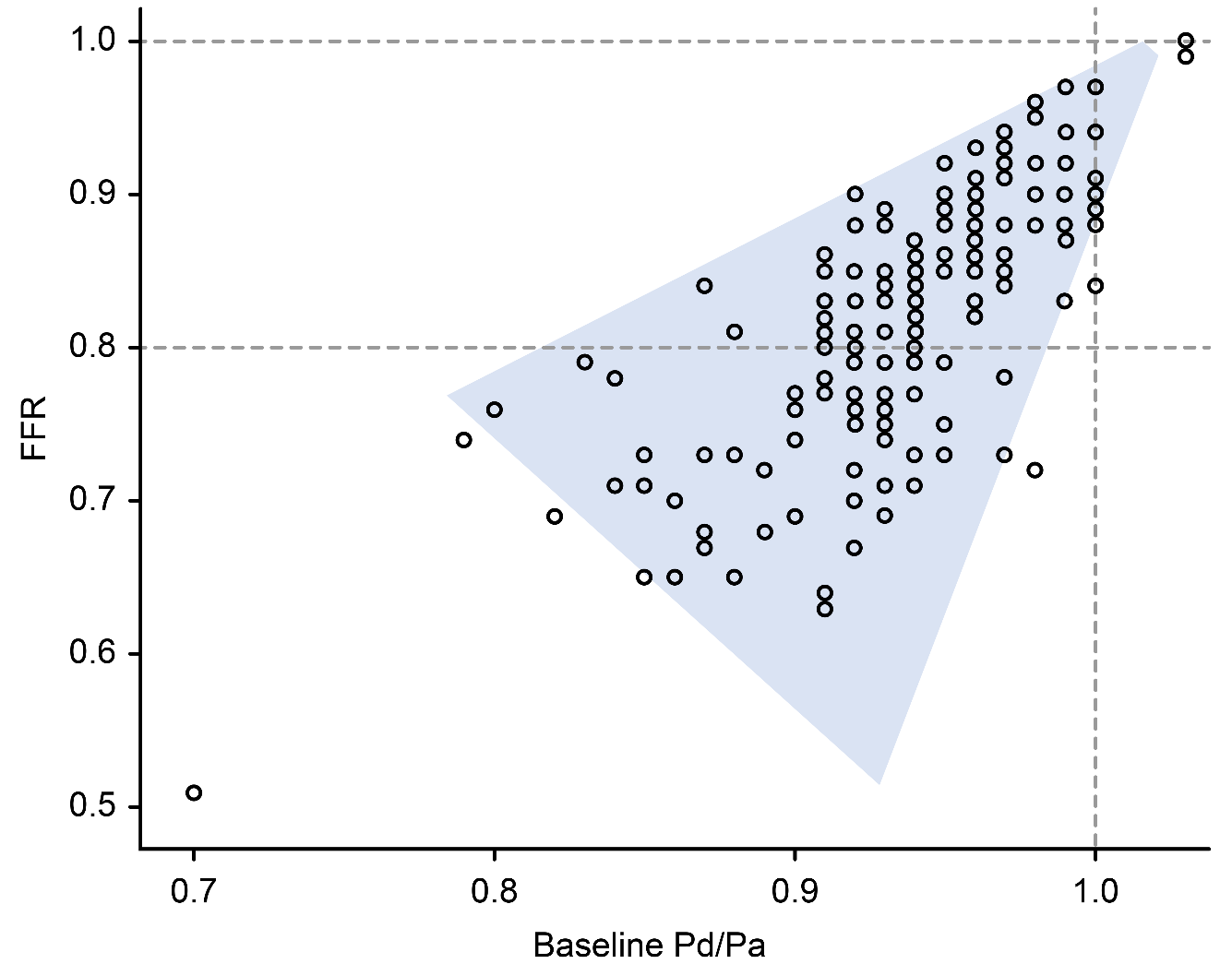
**

**Supplementary Figure S1. Associations between the baseline Pd/Pa and FFR**

The baseline Pd/Pa and FFR are represented as scatter plots. The baseline Pd/Pa and FFR are represented as triangles in the scatter plots with no added statistical analysis. The scatter plot appears to spread as the baseline Pd/Pa and FFR decrease, with an FFR of 1.0 and baseline Pd/Pa of 1.0 as the point of intersection.

FFR, fractional flow reserve; Pd/Pa, distal-to-aortic pressure ratio.


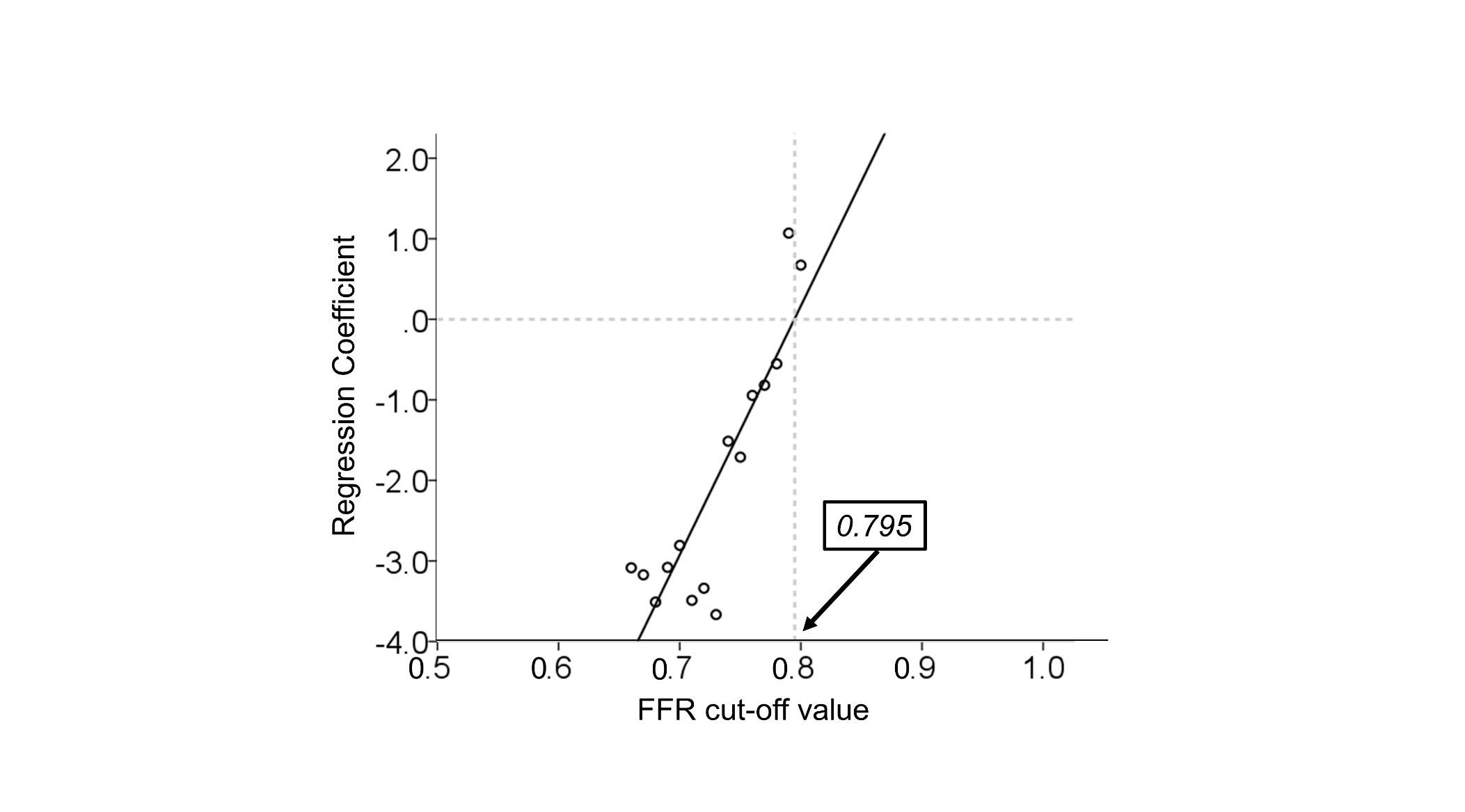


**Supplementary Figure S2. Association between regression coefficient and FFR cut-off value**

The X-axis represents the FFR cut-off value, and the Y-axis represents the regression coefficient between baseline Pd/Pa and LogBNP levels. We found that the FFR at which the slope of the regression line became 0 was 0.795.
